# MicroRNA-34a: the bad guy in age-related vascular diseases

**DOI:** 10.1007/s00018-021-03979-4

**Published:** 2021-10-26

**Authors:** Angela Raucci, Federica Macrì, Stefania Castiglione, Ileana Badi, Maria Cristina Vinci, Estella Zuccolo

**Affiliations:** 1 Experimental Cardio-Oncology and Cardiovascular Aging Unit, Centro Cardiologico Monzino-IRCCS, Via C. Parea 4, 20138 Milan, Italy; 2 Unit of Vascular Biology and Regenerative Medicine, Centro Cardiologico Monzino-IRCCS, Milan, Italy; 3 Division of Cardiovascular Medicine, Radcliffe Department of Medicine, University of Oxford, Oxford, UK

**Keywords:** Senescence, *Inflammaging*, microRNA, Vascular calcification, Atherosclerosis, Diabetes

## Abstract

The age-related vasculature alteration is the prominent risk factor for vascular diseases (VD), namely, atherosclerosis, abdominal aortic aneurysm, vascular calcification (VC) and pulmonary arterial hypertension (PAH). The chronic sterile low-grade inflammation state, alias *inflammaging*, characterizes elderly people and participates in VD development. MicroRNA34-a (miR-34a) is emerging as an important mediator of *inflammaging* and VD. miR-34a increases with aging in vessels and induces senescence and the acquisition of the senescence-associated secretory phenotype (SASP) in vascular smooth muscle (VSMCs) and endothelial (ECs) cells. Similarly, other VD risk factors, including dyslipidemia, hyperglycemia and hypertension, modify miR-34a expression to promote vascular senescence and inflammation. miR-34a upregulation causes endothelial dysfunction by affecting ECs nitric oxide bioavailability, adhesion molecules expression and inflammatory cells recruitment. miR-34a-induced senescence facilitates VSMCs osteoblastic switch and VC development in hyperphosphatemia conditions. Conversely, atherogenic and hypoxic stimuli downregulate miR-34a levels and promote VSMCs proliferation and migration during atherosclerosis and PAH. *MiR34a* genetic ablation or miR-34a inhibition by anti-miR-34a molecules in different experimental models of VD reduce vascular inflammation, senescence and apoptosis through sirtuin 1 Notch1, and B-cell lymphoma 2 modulation. Notably, pleiotropic drugs, like statins, liraglutide and metformin, affect miR-34a expression. Finally, human studies report that miR-34a levels associate to atherosclerosis and diabetes and correlate with inflammatory factors during aging. Herein, we comprehensively review the current knowledge about miR-34a-dependent molecular and cellular mechanisms activated by VD risk factors and highlight the diagnostic and therapeutic potential of modulating its expression in order to reduce *inflammaging* and VD burn and extend healthy lifespan.

## Introduction

Cardiovascular diseases are the predominant cause of death and disabilities worldwide and their frequency increases progressively with advancing age [1, 2]. Vascular diseases (VD), such as atherosclerosis, aortic aneurysm and pulmonary hypertension, and VD-associated complications, like vascular calcification (VC), play a causal role in the majority of cardiovascular diseases [3–6]. Along with aging, numerous other risk factors, like diabetes, hypertension, dyslipidemia and hyperphosphatemia, contribute to the onset and progression of VD [7–12]. Interestingly, both aging and the aforementioned risk factors share numerous pathological mechanisms, such as cellular senescence and the onset of a low-grade systemic inflammation in the absence of specific insults or an overt infection, defined as *inflammaging* [13–16].

**Fig. 1:**
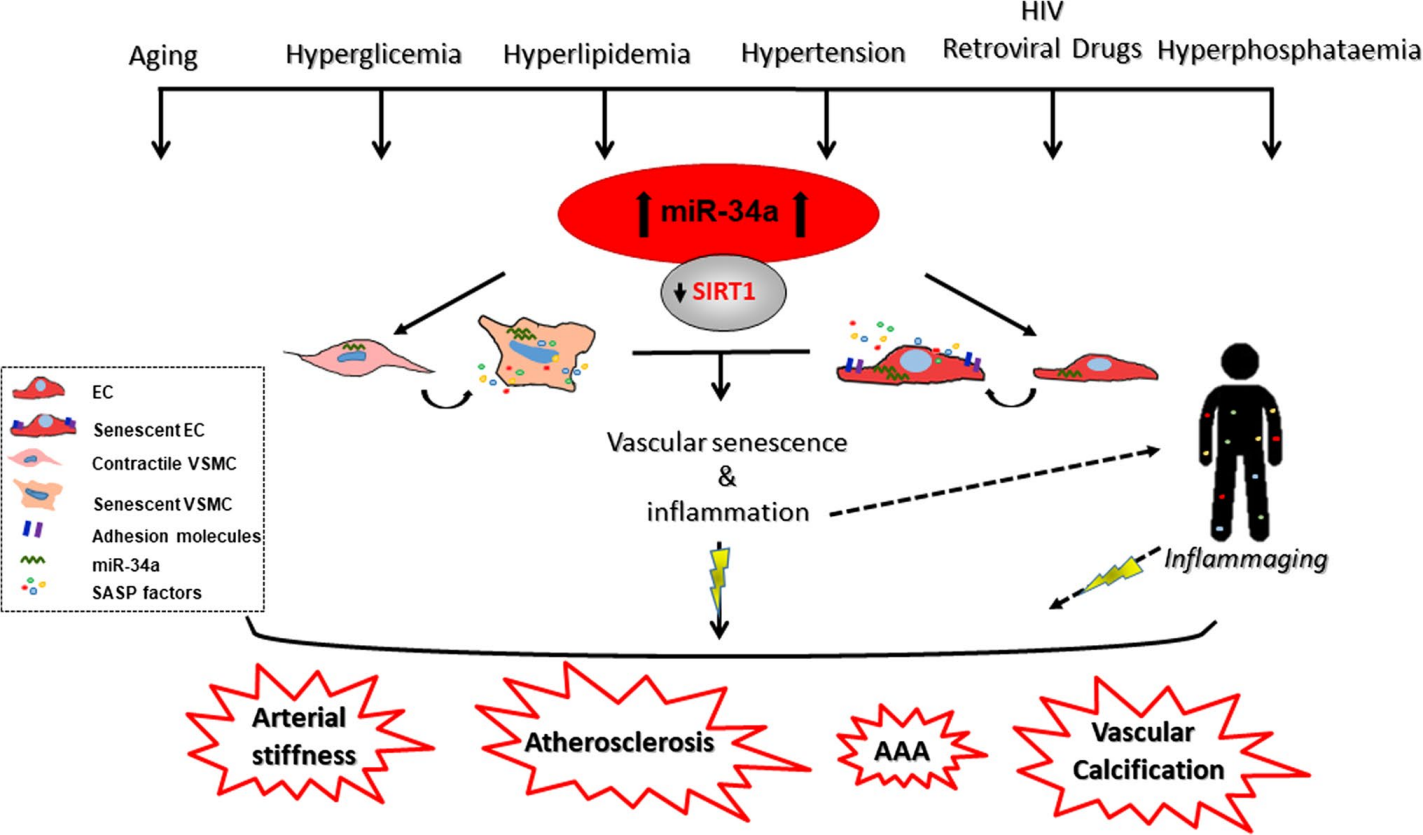
miR-34a involvement in age-associated vascular diseases. Several cardiovascular risk factors, including aging, hyperglycemia, hyperlipidemia, hypertension, hyperphosphatemia as well as HIV-associated proteins and retroviral drugs induce miR-34a expression that by targeting the sirtuin 1 (SIRT1) induces senescence and the acquisition of a senescence associated secretory phenotype (SASP), consisting in the secretion of pro-inflammatory factors, in both vascular smooth muscle cells (VSMCs) and endothelial cells (ECs). Vascular senescence and inflammation contribute to arterial stiffness and facilitate the onset of vascular diseases (VD), such as atherosclerosis, abdominal aortic aneurism (AAA) and vascular calcification. SASP factors produced by senescent vascular cells, by acting in a paracrine and autocrine manner, spread senescence and promote arterial and systemic *inflammaging* increasing the risk of VD development

MicroRNAs (miRNAs) are small non-coding single-stranded RNAs of 20–24 nucleotides that can bind to 3’-untranslated regions (UTRs) of messenger RNAs to either inhibit their translation or induce degradation and, thereby, acting as negative post-transcriptional regulators of gene expression [17]. miRNAs may also enhance messenger RNA translation, depending on the cell cycle phase [18]. miRNAs are implicated in tissue development, regeneration and aging, as well as in the onset and progression of several diseases, including VD [19–22]. Moreover, they represent a promising class of therapeutic targets and useful diagnostic and prognostic biomarkers [23, 24]. In the field, microRNA-34a (miR-34a) was firstly described as a p53-induced tumor suppressor miRNA able to control cell proliferation, apoptosis and senescence of tumor cells [25–27]. More recently, studies documented miR-34a as an important regulator of age-dependent tissues changes and a cell senescence inducer. miR-34 loss-of-function mutation in *Caenorhabditis elegans* delays the age-related physiological decline and prolongs lifespan [28]. miR-34a levels increase with age in several mammalian organs including muscle, heart and aorta, and in some cases indorses detrimental organ remodeling and functional impairment [29–32]. In the vascular wall, miR-34a promotes endothelial cells (ECs) and vascular smooth muscle cells (VSMCs) senescence mainly through the direct downregulation of its most known target, the longevity-associated gene sirtuin 1 (SIRT1; [29, 31]), and in this way supports arterial inflammation and the development of age-related VD [29, 31, 33–35]. Besides, miR-34a may be modulated by other risk factors that stimulate vascular changes similar to aging (Fig. 1).

In this review, we will provide a brief overview on vascular aging, senescence and miR-34a regulation and function. Moreover, we will describe the most recent literature on the involvement of miR-34a in VD and -associated complications induced by advancing age and other risk factors that occur during late life.

## Brief remarks on aging and senescence

Aging is characterized by a progressive unavoidable tissue and organ function decline that increases vulnerability to death, and therefore, it represents the principal risk factor for human pathologies, like cancer, cardiovascular diseases, diabetes and neurodegenerative diseases also named as age-related diseases [36]. Several cellular and molecular hallmarks have been identified to define the mammalian aging phenotype, including genomic instability, telomere shortening, epigenetic alterations, proteostasis loss, nutrient sensing deregulation, stem cell exhaustion, mitochondrial dysfunction and cellular senescence [36].

**Fig. 2:**
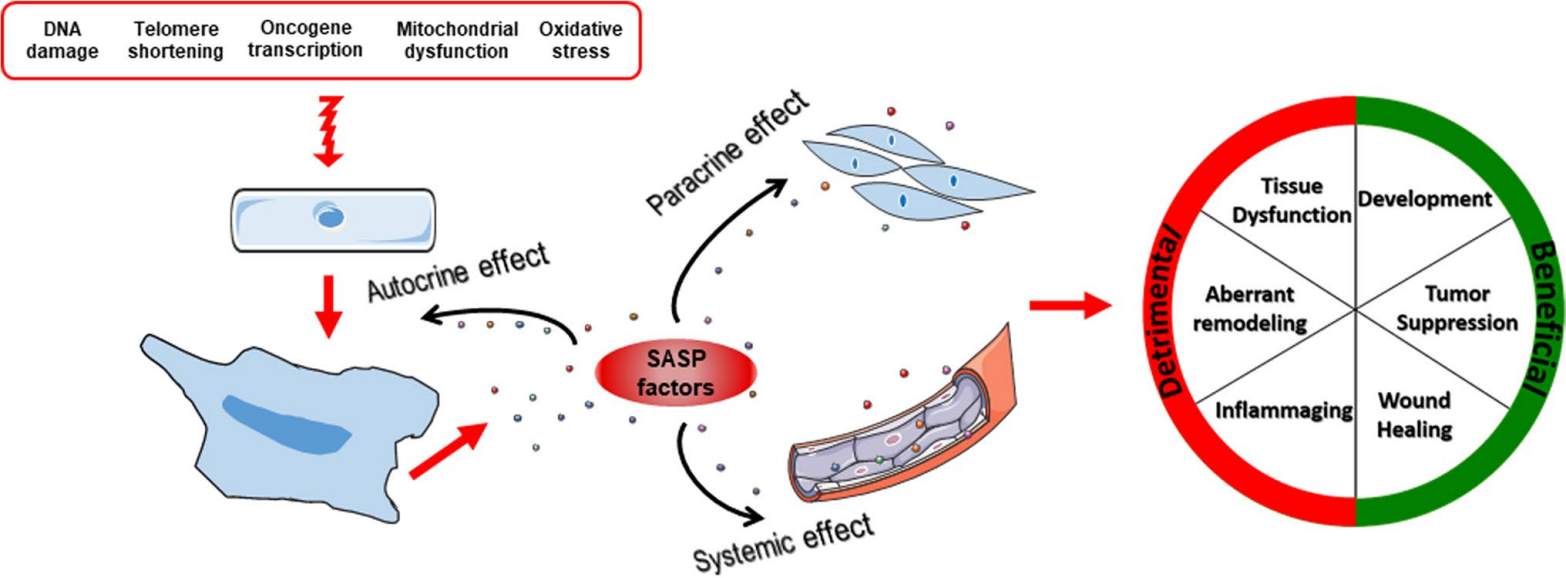
Inducers and features of senescence. DNA damage, telomere shortening, oncogene activation, oxidative stress and mitochondrial dysfunction are the major inducers of senescence. Senescent cells exhibit a series of distinct biochemical and morphological hallmarks that discriminate them from normal cells including enlarged nuclei and flattened cytoplasm. Senescent cells are also able to produce the so-called senescence-associated secretory phenotype (SASP) factors that include inflammatory molecules, extracellular matrix remodeling enzymes and growth factors. Overall, SASP molecules spread and boost senescence in a paracrine manner on surrounding cells leading to tissue dysfunction and aberrant remodeling, and by entering into the blood stream contribute to *inflammaging*. Cellular senescence may also elicit beneficial effects particularly evident during embryonic development, tumor suppression and tissue repair

Cellular senescence is a stress response that evolves to an irreversible loss of the proliferative ability of cells despite the ideal growth conditions [37]. There is not a universal marker to identify the senescent phenotype that may be highly heterogeneous being influenced by different triggers and the specific cell type. Generally, senescent cells are characterized by high resistance to apoptosis, an enlarged flat morphology, the presence of nuclear senescence-associated heterochromatin foci, lipofuscin and senescence-associated-β-galactosidase enzyme accumulation, and increased expression of the cyclin-dependent kinase (CDK) inhibitors p16 and p21 and the tumor suppressor p53 [37]. Last of all, senescent cells remain metabolically active and develop the so-called “senescence-associated secretory phenotype (SASP),” consisting in the secretion of pro-inflammatory molecules such as interleukin-6 (IL6) and IL8, extracellular matrix (ECM) remodeling enzymes, growth factors and soluble receptors that, acting in a paracrine manner, influence neighboring cells behavior altering the microenvironment ([37, 38]; Fig. 2). Senescence may be driven by different inducers of nuclear DNA damage, telomere shortening, oncogene activation, oxidative stress and mitochondrial dysfunctions and have both beneficial and detrimental consequences depending on the biological context ([37]; Fig. 2). Specifically, early in life, senescence occurs to sustain proper embryonic development and later to suppress tumor expansion and promote wound healing and reparative tissue remodeling after an injury [37]. As drawbacks, senescent cells accumulate with advancing aging causing tissue dysfunction and inappropriate remodeling and eventually favor age-related diseases development [15, 39]. Senescence of stem and/or progenitor cells driven by aging or CV risk factors also impairs tissues regenerative potential [37]. Besides, SASP factors produced by accumulated senescent cells contribute to establish and nourish a state of systemic chronic low-grade inflammation, alias *inflammaging*, typical of older people, that represents an additional risk factor for age-related diseases onset and progression (Fig. 2; [15, 37]).

## Vascular aging and senescence

ECs and VSMCs are the main cell types constituting vessels, namely, arteries and veins. ECs constitute the endothelium, a bioactive and permeable barrier of the blood vessel lying on a basal lamina that covers a sub-endothelial layer of thin connective tissue with sparsely cellular matrix and internal elastic membrane, named intima. VSMCs are elongated spindle-shaped cells chained together by the ECM components, like collagens and elastin, compose the tunica media and are responsible for the contraction and relaxation of the vessel tube. During aging the vasculature undergoes pathophysiological changes, even in the absence of conventional cardiovascular diseases risk factors, with gradual alterations of the tissue structure and function. Aged arteries tend to be stiffer and exhibit medial thickening due to elastin fragmentation, excessive deposition of collagens and VSMCs loss with consequent reduced vascular compliance [8, 40]. Increasing vascular stiffness and endothelial dysfunction, measured as carotid-femoral pulse way velocity and endothelium-dependent dilation, respectively, are independent predictors of cardiovascular events and the major links between physiologic vascular aging and development of cardiovascular diseases, such as atherosclerosis and VC, in older individuals [8]. The main mechanisms responsible for age-related arterial dysfunction are vascular oxidative stress and inflammation [8]. In the endothelium, oxidative stress caused by inadequate antioxidant defense and elevation in reactive oxygen species (ROS) quenches the endothelial production/availability of the vasodilator nitric oxide (NO) leading to deleterious changes in vascular reactivity and hence reduced endothelium-dependent dilation [41]. In aged arteries, inflammation is due to the presence of infiltrated macrophages and lymphocytes in the adventitia that along with vascular cells produce inflammatory factors through NF-κB activation [8]. Both oxidative stress and inflammation affect VSMCs migration and proliferation and, by altering the balance between MMPs and their inhibitors, promote ECM remodeling responsible for age-induced arterial stiffness [40]. Pathways involved in the age-dependent arteries modifications include the mammalian target of rapamycin (mTOR), AMP-activated protein kinase (AMPK), Nuclear factor erythroid 2-related factor 2 (NRF2), SIRT1 and klotho [8].

With aging, arterial tissue accumulates senescent ECs and VSMCs that have been found also in atherosclerotic plaques, abdominal aortic aneurism and in the vasculature of other cardiovascular conditions [42–46]. Senescent ECs nourish inflammation and oxidative stress by increasing the expression of adhesion molecules that support the infiltration of immune cells in the perivascular tissue and produce more mitochondrial ROS [8, 40, 47]. Thanks to a complex transcriptional machinery, VSMCs retain a remarkable plasticity [48]. Aging and other cardiovascular risk factors drive the VSMCs switch from a quiescent differentiated contractile phenotype, typical of a healthy vessel, to a synthetic dedifferentiated one characterized by increased proliferation, migration, altered synthesis of ECM components and the acquisition of a senescent and osteo/chondrogenic-like phenotype [40]. These alterations are driven by several extracellular factors, including Platelet-derived growth factor (PDGF), Transforming Growth Factor β (TGF-β), integrin ligands and Angiotensin II (Ang II; [40]). Besides, both senescent ECs and VSMCs produce inflammatory SASP molecules, such as IL6, IL8, IL1 and monocyte chemoattractant protein-1 (MCP-1), matrix remodeling factors, such as Matrix metalloproteinase (MMP9) and MMP2, and ROS spreading inflammation, oxidative stress, and senescence to neighboring cells of the vascular wall, thus fueling vascular dysfunction as well as systemic inflammation [8, 49]. Genomic instability, reflecting accumulation of DNA damage induced by ROS increase and mechanical stress, is the mechanism underlying senescent cells in the arterial tissue [8, 50]. The detrimental effect of senescence to age-associated vascular complications has been provided by recent findings showing that genetic depletion or pharmacologic clearance with senolytics of p16INK4A-positive cells extends healthy life span and improves vascular remodeling and function in different experimental models of VD [45, 46, 51, 52].

## mIR-34a: general information

miR-34a belongs to the three-member miR-34 family, which includes miR-34a, encoded by its own transcript, and miR-34b and miR-34c, which share a common primary transcript. These three family members have different expression patterns, for instance, in mouse miR-34a is expressed at highest levels in the brain, whereas miR-34b/c in the lungs [53]. Moreover, a bioinformatics analysis revealed that miR-34a supposedly represses a significant lower number of genes compared to the entire miR-34 family [54]. Thus, their tissue-specific function may not be so redundant.

miR-34a is a p53 transcriptional target that is silenced or deleted in several types of cancer and its ectopic expression in cancer cells induces cell cycle arrest, apoptosis and senescence [55–58]. Accordingly, many miR-34a targets are cell cycle regulators and cell proliferation proteins, such as CDK4, CDK6, Cyclin D1, Cyclin E2, E2F1, E2F3 and Cell Division Cycle 25A [55, 57, 59, 60]. However, despite this evidence suggests miR-34a as a *bona fide* critical mediator of p53 and thus as a potential tumor suppressor, its effect on p53 response is rather complex. While Okada and colleagues observed that the functional importance of a positive feedback loop between p53 and miR-34a in tumor suppression could be solely evident in vivo upon p53-haploinsufficiency (only miR-34a ablation along with p53 heterozygosity significantly promoted oncogenesis in a mouse model of lung adenocarcinomas), Rokavec and collaborators showed that the sole *Mir34a* deletion was sufficient to enhance tumor invasion in a colitis-associated intestinal cancer mouse model, thus providing the first genetic evidence of a tumor suppressor function for miR-34a [27, 61]. The work of Navarro and Lieberman underlines the complicated functional relationship between miR-34a and p53, since they suggested that this miRNA may affect multiple target genes that are either negative or positive regulators in the p53 network; moreover, like Conception and colleagues, they proposed that miR-34a is dispensable for p53 function [25, 62]. Furthermore, Samuel and collaborators published a gene expression analysis on non-transformed cell lines that underlines the overlapping but also autonomous roles of miR-34a and p53 in cellular homeostasis [63].

### miR-34a regulation

miR-34a levels are regulated through different mechanisms spanning from epigenetic and transcriptional regulation to its maturation and activity modulation. Indeed, miR-34a expression has been described to be modulated by methylation of its promoter in a broad range of cancers and other diseases, such as preeclampsia and alcoholic liver disease [64–69]. miR-34a expression can also be regulated by histone modifications [70, 71], for instance, Wang and collaborators reported that the long non-coding RNA Lnc34a, that modulates colon cancer stem cells self-renewal, is upregulated in late-stage colorectal cancer where it can epigenetically silence miR-34a by recruiting DNA (cytosine-5)-methyltransferase 3A (DNMT3A) and Histone deacetylase 1 (HDAC1) that respectively and simultaneously methylate and deacetylate this miRNA promoter [72].

Several transcription factors have been described as modulators of miR-34a expression. miR-34a most investigated activator is p53 [53, 56, 73]; although other p53 family members, such as p63 and Tap73, can recognize the same binding sites on miR-34a promoter, they can trigger different biological effects, possibly depending on the stimuli and the cellular context [74, 75]. miR-34a has also been described to be regulated by other transcription factors, such as the ETS family member ELK1, the triiodothyronine nuclear receptor SNAIL, ZEB1, STAT3 and HSF1 [61, 70, 76, 77]. Interestingly, Navarro and colleagues described an alternative phorbol ester-responsive promoter that is located approximately 20 kb upstream of the previously described one and that drives the expression of a longer pri-miR-34a transcript [78]. p53 and SIRT1, insulin-like growth factor-1, the tumor suppressor BRCA1 and preeclampsia have been shown to affect miR-34a biogenesis [65, 79, 80].

Modulation of miR-34a activity has been described by Salzam and collaborators: under normal condition mature miR-34 exists in the cell in an inactive form; in response to DNA damage, it is phosphorylated at its 5’-end, so that it can be loaded into Ago2 and thus become active. This novel mechanism of the miRNA activity regulation is p53-independent and occurs faster than the canonical p53-mediated transcription and processing [81]. Finally, competing endogenous RNA, such as circular RNAs, pseudogene transcripts and long non-coding RNAs can sponge miR-34a in order to promote the expression of certain target genes of this miRNA [82, 83].

### miR-34a function

Since its discovery, miR-34a has been mainly investigated in the field of cancer biology because of its clear function of tumor suppressor in vitro. Indeed, it has been shown to affect several processes that play a crucial role in cancer development, such as cell cycle progression, senescence, apoptosis, self-renewal, differentiation, epithelial to mesenchymal transition, migration, and metastasis [61]. miR-34a can indeed induce cell cycle arrest, mainly, but not only, by blocking G1- to S-phase transition, and inhibit proliferation in different cell types by negatively regulating the expression of several factors that are part of the cell cycle machinery [55, 57, 59, 60, 84–87]. Moreover, these functions are reinforced by positive feedback loops, such as the miR-34a/MDM4/p53 or the miR-34a/SIRT1/p53 ones [27, 88]. These pathways are involved not only in the mere cell cycle regulation but also in triggering senescence and apoptosis. In fact, miR-34a affects both replicative and premature senescence. This miRNA expression levels increase during replicative senescence in several cell types as well as during aging in many organs and tissues of humans and mice [29–31, 89–92]; furthermore, miR-34a inhibition results in an extension of the replicative lifespan in late-passage fibroblasts [89]. miR-34a has been suggested to modulate replicative senescence through the negative regulation of targets, such as SIRT1, E2F and thioredoxin reductase 2 [31, 57, 93]. This miRNA is also upregulated during B-RAF oncogene-induced senescence, where it targets the proto-oncogene MYC and we and others demonstrated that miR-34a enhances oxidative stress-induced premature senescence via the inhibition of SIRT1 [29, 76].

Several studies have demonstrated the pro-apoptotic function of miR-34a [53, 94, 95]. However, a few groups were not able to observe the expected miR-34a-induced apoptosis in their experimental conditions, suggesting that this miR-34a-mediated process is cell context dependent [57]. Despite the abovementioned critical functions in cancer, that are underlined by the fact that MRX34 (a double-stranded miR-34 mimic encapsulated in a liposomal nanoparticle formulation) was the first miRNA mimic to enter Phase I clinical trial for primary liver cancer, other selected solid tumors, and hematologic malignancies (ClinicalTrials.gov Identifier: NCT01829971; the trial was closed early due to serious immune-mediated adverse events that resulted in four patient deaths [96, 97]). miR-34a affects other processes, such as stem cell differentiation, neuronal development, aging and cardiovascular functions [30, 90, 98–102]. Hence, miR-34a has been implicated not only in cancer but also in other diseases, such as brain disorders, obesity and cardiovascular diseases [30, 54, 103–106].

## mIR-34a in VD and-associated complications

### miR-34a in vascular aging and senescence

*Mir34a*^*−/−*^ mice do not display any gross vascular developmental defect [25, 107], nevertheless young animals show a basal higher aortas medial thickness and cellular density than Mir34a^+/+^ mice and *Mir34a*^*−/−*^-derived smooth muscle cells exhibit a higher proliferation rate compared to wild-type cells suggesting that in youth miR-34a may act as a brake to regulate proper vascular homeostasis [42]. miR-34a levels increase in aged murine aortas along with p16 and p21 expression and the SASP factor IL6 [35, 42], indicating a feasible causal role of this miRNA in arterial senescence and *inflammaging* and hence in VD. To this regard, miR-34a has been found to regulate both ECs and VSMCs senescence and SASP ([31, 35, 42]; Fig. 1).

**Fig. 3:**
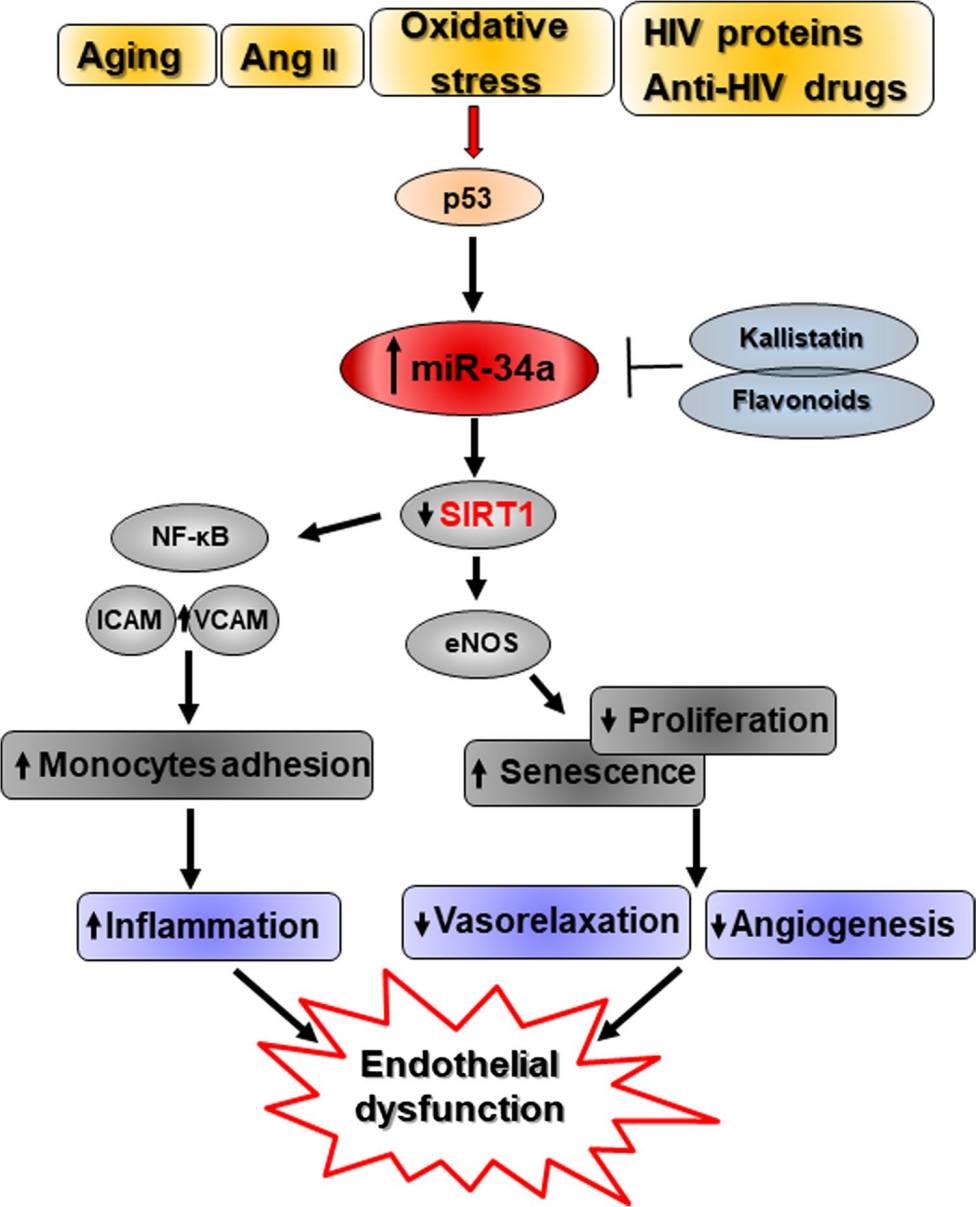
Molecular mechanisms regulated by miR-34a in endothelial cells contributing to endothelial dysfunction. Several stimuli, like aging, Angiotensin II (Ang II), oxidative stress, human immunodeficiency virus (HIV) proteins and antiretroviral drugs, enhance p53-mediated miR-34a expression in Endothelial (ECs) or Endothelial Progenitor (EPCs) Cells. miR-34a increase inhibits ECs/EPCs proliferation and promotes their senescence by targeting the sirtuin 1 (SIRT1) that reduces nitric oxide (NO) availability by downregulating endothelial NO synthase (eNOS). In turn, this leads to impaired angiogenesis and arterial vasorelaxation. miR-34a-targeting of the SIRT1 also augments arterial inflammation by promoting the expression of adhesion molecules, like Vascular Cell Adhesion Protein 1 (VCAM1) and Intercellular Adhesion Molecule 1 (ICAM1), that facilitates monocytes recruitment and invasion through NF-κB activation. Altogether, modulation of these pathways promotes endothelial dysfunction and contributes to VD development and advance. In red are highlighted known direct targets of miR-34a

miR-34a is highly expressed in ECs, including human umbilical cord endothelial (HUVEC) and human aortic endothelial cells (HAEC), and its levels further enhances during replicative senescence [31, 108] or after senescence stimuli, like Ang II and oxidative stress ([109, 110]; Fig. 3). miR-34a overexpression inhibits cell cycle progression of human and murine arterial ECs and HUVEC and promotes senescence through the direct downregulation of SIRT1 [111]. miR-34a also favors endothelial progenitor cells (EPCs) senescence by suppressing SIRT1 and the endothelial Nitric Oxide Synthase (eNOS) expression [92, 112]. Endothelial progenitor cells are bone marrow-derived ECs precursors that play an important role in maintaining endothelial integrity and whose aging acceleration and functional abnormalities have been associated with several VD [113]. Hence, miR-34a increase may impair endothelial homeostasis and renewal at different levels.

Recent studies indicate that both immunodeficiency virus (HIV)-associated proteins and antiretroviral therapy accelerate vascular aging by promoting endothelial senescence and dysfunction and atherosclerotic lesions. Nowadays, these complications represent the major cause of death in HIV population mainly because of the increased survival of patients under highly active antiretroviral therapy [114–116]. miR-34a levels were found increased in arterial vessels and ECs derived from HIV-infected subjects and/or HIV patients taking antiretroviral drugs. Treatment of ECs with recombinant HIV-associated viral proteins (Tat or the envelope glycoprotein gp120) enhances its synthesis as well as cell senescence via p53 activation and ECs isolated from *Mir34a*^*−/−*^ mice show resistance to antiretroviral agents- and HIV-Tat protein-induced senescence due to SIRT1 downregulation blocking [111, 117]. Accordingly, HIV-1 Tat transgenic mice or animals undergone antiviral therapy display miR-34a levels enhancement and administration of an AntagomiR-34a ameliorates aortic endothelial dysfunction [111].

Fan and colleagues published that miR-34a is involved in the flow-dependent regulation of endothelial inflammation by affecting Vascular Cell Adhesion Protein 1 (VCAM1) and Intercellular Adhesion Molecule 1 (ICAM1) expression and consequently monocyte adhesion pinpointing the inflammatory role of this miRNA [34].

Interestingly, several natural compounds and endogenous molecules have been shown to exert an anti-senescent protective action by impairing miR-34a synthesis. Kallistatin, a tissue kallikrein-binding protein and a serine proteinase inhibitor, prevents oxidative stress-induced senescence and inflammation by upregulating Let-7 g that, in turn, inhibits miR-34a synthesis and stimulates SIRT1-eNOS pathway in ECs and EPCs [109, 112]. Similarly, natural flavonoids isolated from *C. cathayensis* Sarg leaves protects HUVEC from Ang II‐induced senescence through inhibition of miR‐34a synthesis and upregulation of SIRT1 expression ([110]; Fig. 3).

**Fig. 4:**
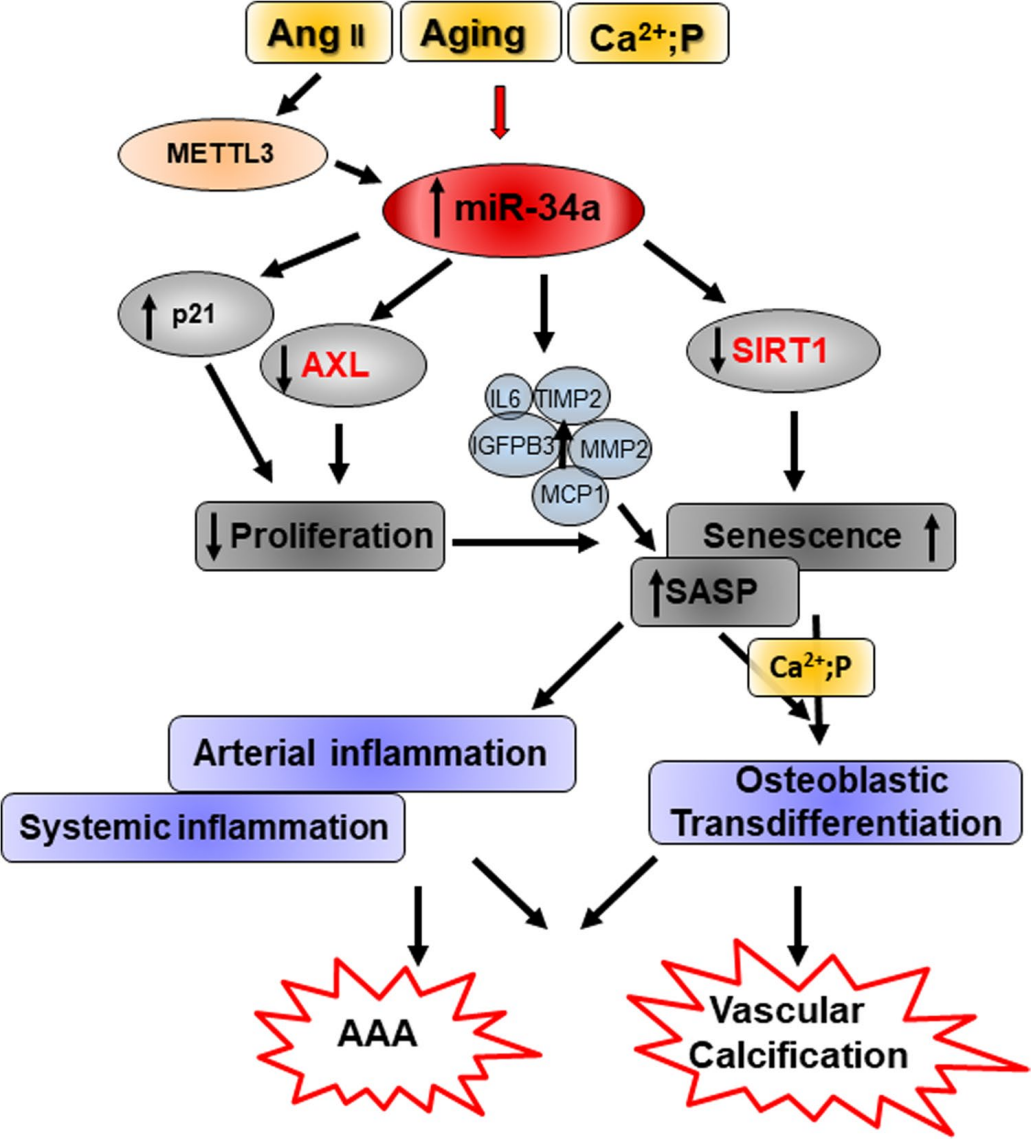
Molecular mechanisms regulated by miR-34a in vascular smooth muscle cells leading to Vascular Calcification and Abdominal Aortic Aneurism. Different stimuli, such as aging, Angiotensin II (Ang II), and hyperphosphatemia (high levels of calcium and phosphate, Ca^2+^; P), are able to induce miR-34a levels in vascular smooth muscle cells (VSMCs). miR-34a increase inhibits VSMCs proliferation via upregulation of p21 and direct downregulation of the receptor tyrosine kinase AXL and promotes VSMCs senescence by targeting the sirtuin 1 (SIRT1). miR-34a also influences VSMCs SASP acquisition by promoting the secretion of specific SASP factors, including IL6, and in this way favors vascular and systemic inflammation and senescence spreading. Senescent VSMCs are more prone to switch to an osteoblastic-like phenotype responsible for vascular calcification onset. Angiotensin II (Ang II) induces miR-34a levels through the induction of Methyltransferase-like 3 (METTL3) expression that enhances miR-34a maturation in VSMCs and eventually favors abdominal aortic aneurism (AAA) development. In red are highlighted known direct targets of miR-34a

Our group demonstrated that miR-34a expression increases in replicative senescent human aortic smooth muscle cells (HASMCs) and with cell donors’ age [29, 35]. miR-34a overexpression inhibits HASMCs proliferation by blocking the G0/G1-S phase transition via p21 upregulation and by directly targeting the anti-apoptotic receptor tyrosine kinase AXL, while it promotes senescence through SIRT1 downregulation [29, 42]. Furthermore, miR-34a influences VSMCs SASP acquisition and favors vascular senescence spreading. Indeed, miR-34a enhances the secretion of specific SASP factors, such as IL6, IL12, IL13, Growth-Regulated Oncogene-alfa (GRO-α)), Tissue Inhibitor of Metalloproteinases 2 (TIMP2) and the pro-senescent Insulin-like Growth Factor-Binding Protein 3 (IGFBP3; [118, 119]) and preconditioning with miR-34a-induced “secretome” boosts HASMCs senescence and their pathologic trans-differentiation ([35]; Fig. 4). In this way, miR-34a can also contribute to systemic low-grade inflammation onset; accordingly, a positive correlation between circulating miR-34a and IL6 in a population of 20–90-year-old healthy subjects has been reported ([35]; Table 1).

**Table 1:** miR-34a as biomarker of aging and vascular diseases

Patient cohort	Disease	Source	Normalization	Observation	References
128 HC subjects (20–90-year-old)	Aging	Serum	Unspecified spike-in synthetic miRNA	Positive Correlation with IL-6 and not IL-8	[35]
106 subjects (median age 68 years old)	Patients subjected to carotid and femoral endarterectomy or an abdominal aortic bypass and LITA samples obtained during coronary artery bypass surgery	Atherosclerotic plaques	Unspecified spike-in synthetic miRNA	Up-regulated	[142]
32 CAD subjects (50–68 years old); 20 volunteers (47–71 years old)	CAD versus HC	Plasma	U6	Up-regulated	[143]
Patients subjected to carotid endarterectomy	Atherosclerosis	Atherosclerotic plaques	U6	Up-regulated	[144]
50 subjects with primary hypertension (51–82 years old); 28 volunteers	Primary hypertension versus HC	Peripheral blood	Unspecified spike-in synthetic miRNA	Up-regulated correlated with the clinical phase of hypertension	[147]
102 subjects (median age 59 years old)	CAD versus HC	PBMCs	U6	Upregulated independently associated with CAD but associated with aortic stiffening and atherosclerosis	[146]
118 subjects (median age 67 years old)	CAD versus HC	Cultured EPCs isolated from PBMC	U6	Higher levels in CAD vs. non-CAD Effect of statin	[145]
56 subjects	s-NGT, pre-diabetes n-T2D diabetes	Serum	RNU6B	Upregulated in n-T2DM vs. pre-diabetes and/or s-NGT	[183]
Meta-analysis on T2DM subjects	Diabetes	Blood	Microarray	40 dysregulated miRNAs in T2DM, including miR34a	[184]
125 subjects (median age 57 years old)	T2DM versus HC	PBMCs	U6	Upregulated and positively correlated with LDL/HDL and Foxp3 but not with triglyceride/HDL	[185]
133 subjects (median age 62 years old)	T2DM, T2DM with nephropathy, diabetic foot, CVD, pre-diabetes	Serum	Cel-miR-39	Upregulated in n-T2DM vs. pre-diabetes and HC	[186]
92 subjects (median age 45 years old)	T2DM, pre-diabetes, LADA, T1DM versus HC	Plasma	hsa-miR-191 and miR-451	Upregulated in T2DM, and T1DM patients	[187]
60 subjects (median age 31–50 years old)	T2DM versus HC	Plasma	Spike-in-control, cel-miR-39	Upregulated	[188]

*CAD *coronary artery disease, *CVD *cardiovascular disease, *EPCs *endothelial progenitor cells, *HC *healthy controls, *HDL *high-density lipoprotein, *LADA *latent autoimmune diabetes, *LITA *left internal thoracic artery, *n-T2DM *newly diagnosed type 2 diabetes patients, *PBMC *peripheral blood mononuclear cells, *s-NGT *susceptible individuals with normal glucose tolerance, *T1DM *type 1 diabetes

Thus, the age-associated miR-34a upregulation, by inducing senescence and SASP of ECs and VSMCs, represents a crucial promoter of vascular aging as well as arterial and systemic *inflammaging* and, eventually, contributes to the developing of age-dependent VD such as atherosclerosis and VC (Fig. 1).

### miR-34a in vascular calcification

Vascular calcification (VC) is a complex disorder typical of aging, diabetes, renal dysfunction and atherosclerosis characterized by deposition of calcium and phosphate minerals as hydroxyapatite crystals within the wall of major and minor blood vessels with subsequent mineralization of the extracellular matrix of the tissue [3, 11, 120, 121]. VC is one of the main causes of arterial stiffening and reduced compliance and for this reason it is strongly associated with cardiovascular mortality [122, 123].

In the major arteries, calcification may occur in both the intimal and medial layers, while in the coronary arteries occurs primarily in the intimal atherosclerotic plaque [124]. VC shares some common features with bone morphogenesis and recent studies have highlighted VC as an actively cell-mediated process driven primarily by VSMCs reprogramming towards an osteochondrogenic lineage [125, 126]. Likewise, stressed ECs can undergo osteogenic transformation through endothelial–mesenchymal transition [127]. Aging and several other risk factors, like hyperglycemia, hyperlipidemia, dysregulated mineral metabolism (hyperphosphatemia and intermittent hypercalcemia), along with uremic toxins and oxidative stress promote VSMCs shift from a contractile to synthetic phenotype that lose the ability to produce endogenous calcification inhibitors, namely, Osteopontin, Matrix Gla protein and pyrophosphate (PPi). In the presence of persistent stress signals, synthetic VSMCs undergo further maladaptive osteoblastic differentiation and eventually necrosis and death, resulting in hydroxyapatite deposition in the extracellular matrix of the vessel [10, 11, 128, 129]. Besides, senescence and the acquisition of SASP increase the propensity of VSMCs to experience osteoblastic transition [130, 131], indeed senescent VSMCs are characterized by the expression of bone-related genes, Runt-related transcription factor 2 (Runx2), Alcaline Phosphatase and osteocalcin and secretion of pro-calcification SASP molecules like IL-6, bone morphogenetic protein 2 (BMP2) and osteoprotegerin, that are able to induce both senescence and osteoblastic differentiation on neighboring VSMCs and local or circulating stem cells [120, 131–134].

Experimental evidences have emerged suggesting that miR-34a can influence osteoblastic differentiation and bone formation as well as ectopic calcification. miR-34a levels have been shown to increase during osteoblastic differentiation of human mesenchymal stem cells (hMSC) in vitro and in mature osteoblastic cells in vivo [59]. The over-expression of miR-34a inhibits both the switch of the hMSCs towards osteoblastic lineage and the differentiation process itself; moreover, bone formation decreases in vivo [59]. Opposite results have been obtained by Fans and collaborators that observed increased miR-34a levels during the osteogenic differentiation of human fat stem cells (hASCs) and that the over-expression of this miRNA enhances significantly the mineralization of hASCs in vitro and promotes ectopic bone formation in vivo [135]. Recently the expression of miR-34a has been found upregulated in valve tissue from calcific aortic valve stenosis. miR-34a induces calcium deposition in porcine aortic valve interstitial cells in vitro and locked nucleic acid miR-34a inhibitor suppresses mineralization of aortic valves and cardiac hypertrophy in a wire injury calcific aortic valve stenosis mice by modulating Notch1/Runx2 signaling ([136]; Table 2).

**Table 2:** Use of miR-34a antagomir or mimic in experimental animal models of vascular diseases

Animal Model	miR-34a inhibitor	miR-34a mimic	Route of administration	Treatment effect		References
HIV-1 Tat transgenicmice	Antago-miR-34a	–	Tail-vein	Beneficial: ameliorates aortic endothelial dysfunction	–	[111]
Antiretroviral drugs (lopinavir/ritonavir)-treated mice	Antago-miR-34a	–	Tail-vein	Beneficial: ameliorates aortic endothelial dysfunction	–	[111]
Vit D-treated *Mir34a*^*−/−*^ mice	Genetic global ablation	–		Beneficial: reduces soft tissue and aorta calcification; it decreases vascular inflammation	–	[35, 42]
HFD-treated ApoE−/– mice	Antago-miR-34a	–	Tail-vein	Beneficial: abates aortic atherosclerotic plaque lesion development	–	[149]
Femoral artery denudation injury mice		Ago-miR-34a	Perivascular delivery	Beneficial: prevents neointima formation	–	[155]
Western diet fed Ldlr–/– or ApoE–/– mice		Hepatic overexpression of miR-34a by an adenovirus			Detrimental: causes liver steatosis. Protective: atheroprotective	[157]
HFD-treated *Mir34a* macrophage-specific or global ablation in Ldlr-/- or ApoE-/- mice				Beneficial: improves dyslipidemia, reduces atherosclerosis, obesity and NAFLD		[144]
HFD-treated Ldlr-/- or ApoE-/- mice	LNA miR-34a inhibitor		Systemic	Beneficial: improves dyslipidemia, obesity and NAFLD and reduces atherosclerosis		[144]
AAA induced by Ang II infusion in ApoE-/- mice	AAV9-anti-miR-34a	AAV9-miR-34a	Systemic	Beneficial: Inhibits Ang II-induced AAA formation, vascular senescence and inflammation	Detrimental: Exacerbates Ang II-induced AAA formation, vascular senescence and inflammation	[167]
Hypoxia-induced PAH in rats		miR-34a mimic	Intratracheal nebulization		Prevention of hypoxia-induced pulmonary hypertension and pulmonary vascular remodeling	[173]
Type 2 diabetes db/db mice and STZ-induced type 1 diabetes in mice	LNA miR-34a inhibitor		Systemic	Beneficial: preserves endothelium-dependent vasorelaxation without affecting the hyperglycemic status		[195]
Endothelial miR-34a knockout mice				Beneficial: preserves endothelium-dependent vasorelaxation without affecting the hyperglycemic status		[195]
STZ-induced type 1 diabetes in mice	Antago-miR-34a		Subcutaneous injection once a week for 24 weeks	Beneficial: mitigates tunica media thickness and impaired contraction and relaxation		[194]

*AAA *abdominal aortic aneurism, *AAV9 *adeno-associated Virus Serotype 9, *Ang II *Angiotensin II; *ApoE *Apolipoprotein E, *HFD *high-fat diet, *HIV *human immunodeficiency virus, *LNA *locked nucleic acid, *Ldlr *low-density lipoprotein receptor, *NAFLD *non-alcoholic fatty liver disease, *PAH *pulmonary arterial hypertension, *ST *streptozotocin, *Vit D *Vitamin D

Our studies demonstrate that upregulation of miR-34a is necessary to promote VSMCs senescence and SASP that eventually trigger the onset and progression of VC [29, 35]. miR-34a induction precedes aortas mineralization in a mouse model of medial aortic and soft tissues calcification induced by an overdose of Vitamin D [42]. *Mir34a*^*−/−*^ mice exhibit reduced soft tissue and aorta calcification along with decreased expression of p21, p16, Runx2 and SRY (sex-determining region Y)-box 9 (Sox9). SMC isolated from *Mir34a*^*−/−*^ show lower senescence-associated-β-galactosidase activity and p16 expression and less calcium deposition when cultured in an osteogenic medium in comparison to *Mir34a*^+*/*+^ cells [42]. HASMCs overexpressing miR-34a or preconditioned with miR-34a-induced “secretome” display an enhanced mineralization in hyperphosphatemic conditions and show concomitant downregulation of AXL and SIRT1, that are inhibitors of VSMCs mineralization ([35, 42]; Fig. 4). Interestingly, Hao et al. showed that the other two members of the miR-34 family, miR-34b/c, are negative regulators of VSMCs calcification since their over-expression or inhibition of miR-34b/c slow and enhances VC, respectively [137].

Thus, miR-34a is a promoter of VC being responsible for both the cell autonomous senescence-induced VSMCs mineralization and senescence spreading to nearby cells through paracrine signals. Moreover, VC depends on the expression balance of all miR-34 family members that is under the control of different promoters.

### miR-34a in atherosclerosis

Atherosclerosis is an age-related pathology initially considered as a disorder due to altered lipoprotein deposition in the arteries. Nowadays, atherosclerosis represents the major cause of coronary heart disease, such as myocardial infarction, stable and unstable angina and sudden death, and it is considered a vascular chronic disease characterized by increased oxidative stress, inflammation, susceptibility to apoptosis and presence of early cellular senescence [51, 138]. The initial stage of atherosclerotic plaque formation is the chronic endothelial cell injury that leads to upregulation of endothelial adhesion molecules ICAM-1 and VCAM-1 that are responsible for leukocytes recruitment and platelets adhesion, and a greater vascular permeability, responsible for accumulation of lipoprotein, mainly low-density lipoprotein (LDL), in arteries regions where the blood laminar flow is disturbed for the presence of bends or branch points [12]. Subsequently, activated recruited macrophages start to secrete pro-inflammatory cytokines and chemokines and produce free radicals to enhance inflammation, recruit other immune cells, and oxidize the accumulated lipoproteins. After swallowing the oxidized LDL (ox-LDL), macrophages become foam cells and form fatty streaks. The following steps consist in the accumulation of platelets and proliferation, migration from media to neo-intima, and osteogenic trans-differentiation of VSMCs along with deposition of an altered amount of collagens, elastin and proteoglycans. Altogether, these elements generate a fibrous plaque that can obstruct blood flow and, in the worst case, lead to the formation of a thrombus [139]. Lately, the critical role of senescence in atherosclerosis has been recognized. Senescent ECs, VSMCs and macrophages have been found in the plaque and reported to influence all different disease stages and their genetic and/or pharmacologic elimination reduces atherosclerotic plaque formation and progression [45, 52, 140]. ECs senescence contribute to endothelial damage, senescent macrophages exhibit an abnormal activation and senescent VSMCs differentiate in osteogenic-like cells participating to plaque formation and calcification [138, 139]. Moreover, the SASP of senescent cells promotes arterial inflammation facilitating atherosclerosis onset and worsening the outcome [141].

A first evidence of miR-34a involvement in atherosclerosis has been proposed by the Tampere Vascular Study that investigates the expression profile of about 900 miRNAs and their predicted targets in human atherosclerotic plaques by miRNA microarray [142]. miR-34a along with miR-21, -146a, -146b-5p and -21 were found upregulated in atherosclerotic arteries compared to non-atherosclerotic left internal thoracic arteries [142]. Several predicted miR-34a target genes were downregulated, and gene set enrichment analysis evidenced pathways related to intima-media thickening, phenotype of VSMCs and high-density lipoprotein (HDL) and cholesterol modulation [142]. Subsequent studies confirmed increased levels of miR-34a in the blood or atherosclerotic plaques of coronary artery disease (CAD) patients [143, 144]. Of note, statins affect miR-34a/SIRT1 axis. Indeed, Tabuchi and collaborators found that the number of circulating EPCs isolated from peripheral blood mononuclear cells (PBMC) was lower in subjects with CAD compared to subjects without CAD and that SIRT1 was downregulated, whereas miR-34a was upregulated in cells isolated from CAD patients [145]. Notably, patients treated with atorvastatin had markedly decreased miR-34a and increased SIRT1 levels suggesting that the atheroprotective effect of statin may involve miR-34a activity [145]. Very recently, Gatsiou et al. reported an additive role of PBMC miR-34a/b/c expression in human vascular aging and atherosclerosis and in particular with adverse cardiovascular risk profile evidenced by an independent association with aortic stiffening and the presence and extent of atherosclerosis [146]. The association of miR-34a/b/c with CAD was mainly due to SIRT1 [146]. Other risk factors for atherosclerosis alter circulating concentration of miR-34a; Liu et al. showed that miR‑34a was significantly upregulated in the peripheral blood of patients with hypertension and its circulating amount correlated with the clinical phase of hypertension [147].

**Fig. 5:**
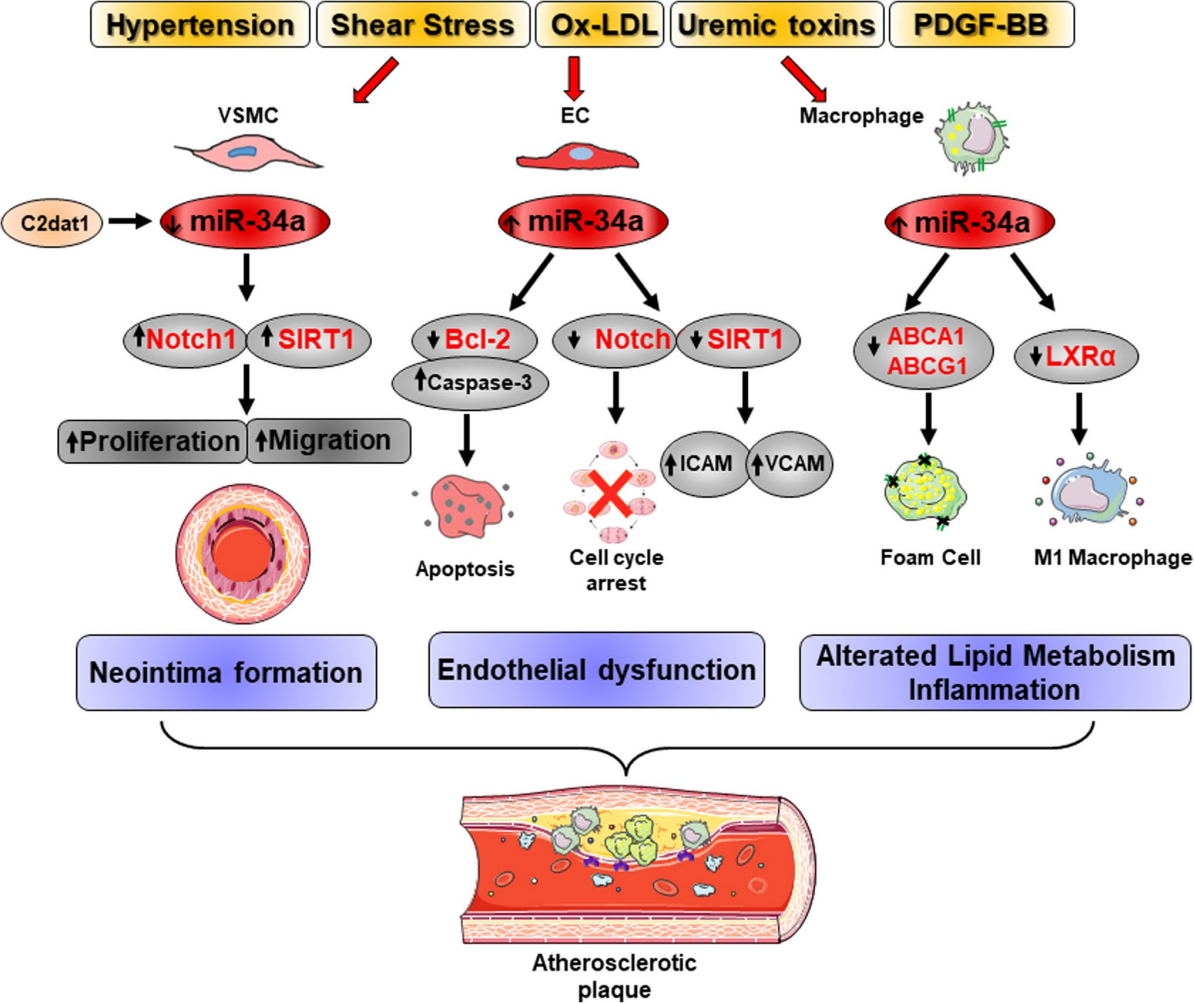
miR-34a in atherosclerosis. Atherogenic stimuli differentially modulate miR-34a expression in vascular cells. miR-34a downregulation due to Platelet-derived Growth Factor B (PDGF-BB), oxidized low-density lipoprotein (ox-LDL) and uremic toxins induces the sirtuin 1 (SIRT1) and Notch1 upregulation that stimulate vascular smooth muscle cells (VSMCs) proliferation and migration and ultimately neointima formation. The long non-coding RNA CAMK2D‐associated transcript 1 (C2dat1) suppresses miR-34a expression in VSMCs. Atheroprone oscillatory shear stress causes miR-34a upregulation and endothelial cells (ECs) activation in terms of increased expression of Vascular Cell Adhesion Protein 1 (VCAM1) and Intercellular Adhesion Molecule 1 (ICAM1) by SIRT1. Similarly, ox-LDL and uremic toxins stimulate miR-34a synthesis and ECs apoptosis and inhibition of their proliferation through B-cell lymphoma 2 (Bcl-2) and Notch1 downregulation, respectively. In macrophages, ox-LDL increases miR-34a levels that target the cholesterol transporters ATP-binding cassette subfamily A member 1 (ABCA1) and ATP-binding cassette subfamily G member 1 (ABCG1) and subsequently reduces macrophages cholesterol efflux capacity and their differentiation in foam cells. Moreover, miR-34a enhances the secretion of inflammatory cytokines by directing M1-type macrophage differentiation through the nuclear hormone Liver X receptor α (LXRα). Altogether, these events alter lipid metabolism and promote inflammation facilitating atherosclerotic plaque formation. In red are highlighted known direct targets of miR-34a

Experimental evidences show that miR-34a is modulated during atherogenesis. Using the animal model of atherosclerosis-prone apolipoprotein E-deficient (ApoE^−/−^) mice fed a western high-fat diet, several groups demonstrated that miR-34a levels increase in the aortas and serum at early and late stages of atherosclerosis [143, 144, 148, 149]. Moreover, higher miR-34a expression has been found in plasma and in platelet-derived microvesicles of hypertensive-hyperlipidemic hamsters, an experimental model of atherosclerosis induced by hypertension [150]. miR-34a may play a causal role in atherosclerosis initiation and progression since it is able to alter ECs and VSMCs functions by inducing their senescence and SASP and by modulating its master targets Notch1 and SIRT1, well-known atheroprotective molecules [41, 51, 151, 152]. Furthermore, Arunachalam and colleagues showed that the NO bioavailability in mouse microvascular endothelial cells (MMEC) operates through a miR-34a-SIRT1 axis-dependent mechanism [33] and inhibition of basal levels of miR‑34a expression promotes HUVEC proliferation and migration and represses apoptosis [147]. With the regard to specific atherogenic insults, Fan et al. reported that miR-34a expression can be modulated by shear stresses in ECs, in particular, the atheroprotective physiological uniform laminar shear stress can downregulate miR-34a level, whereas the atheroprone oscillatory shear stress causes its upregulation and endothelial activation [34]. Ox-LDL induces miR-34a synthesis in HUVEC and HAEC and, thereby, inhibits proliferation and promotes apoptosis and ROS production by directly reducing the expression of the anti-apoptotic target gene B-cell lymphoma 2 (Bcl-2) that leads to Bcl-2 Associated X-protein (Bax)-Caspase-9-Caspase-3 pathway activation [149, 153]. In accordance, administration of anti-miR-34 in HFD-treated ApoE^−/−^ mice abated the development of aortic atherosclerotic plaque lesions by inhibiting ECs apoptosis through Bcl2 downregulation ([149]; (Table 2). Lastly, Li and colleagues showed that the uremic toxin indoxyl sulfate (IS), which has atherogenic properties, inhibits HUVEC viability, migration and proliferation by upregulating miR-34a that, in turn, targets Notch1 signaling pathway ([154]; Fig. 5).

VSMCs are major contributors to plaque development at all stage of atherogenesis [51, 139]. Cultures of VSMCs derived from plaques show a reduced proliferative capacity and VSMCs proliferation has been recognized as a beneficial event throughout the progression and in the advanced stage of atherosclerosis to stabilize plaque formation; instead, VSMCs senescence promotes plaque instability [51, 139]. Although a direct evidence is missing, it is plausible that age-associated miR-34a upregulation promotes and aggravates atherosclerosis by blocking proliferation and inducing senescence, SASP and the osteoblastic phenotypic transdifferentiation of VSMCs ([29, 35, 42]; Fig. 1). In agreement, a number of key genes known to be upregulated in atherosclerotic plaques are also upregulated in senescent VSMCs and overexpression of miR-34a enables HASMCs to express inflammatory SASP molecules some of which are involved in plaque formation [29, 35]. Additionally, miR-34a is downregulated upon multiple atherogenic stimuli, such as Platelet-derived Growth Factor B (PDGF-BB), ox-LDL and IS in murine and human aortic SMC in vitro as well as in a mouse model of wire injury-induced neointimal formation of femoral arteries [154, 155]. Conversely, its overexpression reduces atherogenic stimuli-induced proliferation, migration and apoptosis of these cells by repressing Notch1; accordingly, perivascular delivery of miR-34a AgomiR prevents neointima formation by inhibiting intimal VSMCs proliferation and Notch1 expression in vivo ([155]; Table 2). Finally, the Calcium/calmodulin-dependent Protein Kinase type II subunit delta (CAMK2D)-associated lncRNA and the CAMK2D-associated transcript 1 (C2dat1) suppresse miR-34a expression and promote VSMCs proliferation and migration by inducing SIRT1 expression ([156]; Fig. 5).

An important component of atherosclerosis progression is the alteration of lipid metabolism and the deposition of a fatty streak within the intimal layer of the arteries due to accumulation of lipid-engorged foam cells. miR-34a has been shown to regulate lipid and lipoprotein metabolism at different levels. Xu et al. reported that acute hepatic overexpression of miR-34a in Low-density lipoprotein receptor (Ldlr) knockout (Ldlr^−/−^) or ApoE-/- mice fed a HFD is atheroprotective but causes liver steatosis ([157]; Table 2). Mechanistically, liver-specific overexpression of miR-34a reduces levels of the Hepatocyte Nuclear Factor 4a (HNF4a), a liver-enriched nuclear hormone receptor, resulting in a decrease of very LDL (VLDL) secretion and consequent plasmatic hypolipidemia and reduced aortic root lesions formation [157]. The same group has recently published a study reporting the central role of miR-34a in regulating macrophages cholesterol efflux, polarization and inflammation during atherosclerosis progression [144]. Treatment of murine macrophages with cholesterol, ox-LDL, and inflammatory cytokines increased miR-34a synthesis that, in turn, directly targets the protein expression of cholesterol transporters ATP-binding cassette subfamily A member 1 (*ABCA1*) and ATP-binding cassette subfamily G member 1 (*ABCG1*) 3’ UTR activity. Macrophages isolated from *Mir34a*^*−/−*^ mice reveal an increased *ABCA1* and *ABCG1* expression and cholesterol efflux capacity along with reduced secretion of inflammatory cytokines and M1 markers while an increased M2 markers under the control of the nuclear hormone Liver X receptor α (LXRα) (Fig. 5). In vivo, macrophages-selective *Mir34a* ablation in ApoE^−/−^ mice under a HFD attenuates atherosclerotic aortic lesion formation by reducing monocytes infiltration and local inflammation and promotes cholesterol efflux without affecting hepatic or plasmatic lipid profile [144]. Notably, *Mir34a* global ablation or its pharmacologic inhibition by a locked nucleic acid (LNA) in Ldlr^−/−^ or ApoE^−/−^ animals improves dyslipidemia and reduces not only atherosclerosis but also obesity and non-alcoholic fatty liver disease onset by inducing energy expenditure and coordinating regulation of macrophages cholesterol efflux, hepatic cholesterol uptake and fat and cholesterol intestinal adsorption. Since global or macrophages-selective *Mir34a* ablation affects atherosclerosis to a similar extent, the authors suggest that macrophages-specific expression of miR-34a may have a more crushing effect in the pathology onset compared to hepatic miR-34a. Hence, miR-34a has a pivotal role in atherosclerosis and associates metabolic disorders ([144]; Table 2). Accordingly, several findings suggest that miR-34a may be a culprit for obesity-related cardiometabolic diseases acting on multiple gene targets and in different organs [103, 105, 158–160].

In contrast to these data, Zhao et al. proposed a protective role for miR-34a in atherosclerosis induced by homocysteine, an intermediate of methionine metabolism [161]. miR-34a was found downregulated in ApoE-/- mice fed a high-methionine diet, that increases levels of homocysteine in the blood, and in monocyte-derived foam cells treated with homocysteine [162]. Conversely, preservation of miR-34a levels inhibited the accumulation of total and free cholesterol and triglyceride in homocysteine-stimulated foam cells [162].

Hence, these data underline the important role of miR-34a as a regulator, biomarker and target in atherosclerosis onset and progression induced by aging and other risk factors, such as hypertension and obesity.

### miR-34a in abdominal aortic aneurism

Abdominal aortic aneurism (AAA) is an age-dependent life-threatening disease, with a general prevalence in men, consisting in an irreversible enlargement of the abdominal aortic wall [6, 163]. Patients with AAA are commonly asymptomatic and often found accidentally and the aneurysm rupture accounts for a mortality rate exceeding 80% [6]. Arterial hypertension is a risk factor for AAA [163]. The pathological changes leading to AAA consist primarily in the loss of the aortic structural integrity due to inflammatory cells infiltration in the vessel wall, ECM proteolytic degradation by MMPs and VSMCs and ECs dysfunction [6, 164–166]. Vascular senescence plays a major role in AAA pathogenesis. Vascular cells isolated from AAA patients show telomere attrition and DNA damage [43], and vascular senescence, induced by SIRT1 deficiency, accelerates AAA formation [44].

Zhong et al. explored miR-34a role in an experimental model of AAA induced by Ang II-infusion in ApoE-/- mice. Downregulation or overexpression of miR-34a by adenoviral vectors carrying inhibitors or mimics of miR34a, resulted in suppression or exacerbation of Ang II-induced maximal abdominal aortic diameter, elastin degradation and macrophages infiltration, respectively, without affecting blood pressure variations (Table 2). miR34a mediated Ang II-induced abdominal aortas inflammation and senescence through p21 and SASP factors MMP2 and MCP-1 following direct reduction of SIRT1 expression ([167]; Fig. 4). Mechanistically, the authors showed that higher levels of miR-34a associated with AAA depends on the increased expression of the Methyltransferase-like 3 (METTL3) in VSMCs that is able to enhance miR-34a maturation by promoting N6-methyladenosine (m^6^A) RNA methylation through the recognition of Digeorge syndrome critical region gene 8. Accordingly, in vivo, anti-miR-34a administration attenuates AAA formation promoted by METTL3 overexpression [167].

### miR-34a in pulmonary arterial hypertension

Pulmonary arterial hypertension (PAH) is a chronic progressive vascular obstructive disease characterized by high cell proliferation rate and reduced apoptosis of pulmonary artery smooth muscle cells (PASMCs) and is associated with poor outcomes [9, 168]. The pathophysiological scenario of PAH includes excessive vasoconstriction, inflammation, fibrosis, proliferation/apoptosis imbalance, thrombosis, cellular remodeling and increased right ventricular afterload leading to fatal right ventricular failure [169]. Resistance to apoptosis is the most important molecular mechanism leading to the development of pulmonary remodeling. PAH is considered to have a “neoplastic-like” phenotype that is evident also in cultured PASMCs and is promoted by changes in the mitochondrial metabolism [170]. Senescence cells could participate to the pathogenesis of PAH by contributing to vascular remodeling and degeneration through SASP molecules production, which promote inflammation and fibrosis in and around the vessel wall, and by physically obstructing the vascular lumen due to their intrinsic resistance to apoptosis. Accordingly, correlation between high levels of Osteopontin, together with p16 and p21, and the severity of PAH in aged mice has been demonstrated [171].

In 2013, Mizuno et al. reported higher levels of pulmonary miR-34a in a mouse model of pulmonary arterial remodeling induced by chronic hypoxia that results in pulmonary hypertension and right ventricular hypertrophy [172]. However, lower levels of miR-34a, under the control of p53, associate with the exacerbation of pulmonary arterial remodeling, suggesting that the axis p53-miR-34a mitigate over-proliferation of VSMCs in hypoxic conditions [172]. Years later, Wang et.al observed that miR-34a expression levels were specifically reduced in the lung of a rat model of hypoxia-induced PAH compared to normoxic animals and intra-tracheal nebulization of miR-34a mimics prevented pulmonary artery pressure and medial wall thickness increase [173]. In vitro, the overexpression of miR-34a reduced the hypoxia-mediated PASMCs proliferation and migration by directly downregulating the platelet-derived growth factor receptor alpha expression while promotes apoptosis [173]. Also, miR-34a interferes with the hypoxia-dependent reduction of the Potassium Two Pore Domain Channel Subfamily K Member 3 expression that participates in the regulation of plasma membrane resting potential and vasoconstriction by regulating intracellular calcium concentration [173].

Hence, miR-34a contributes to the pathogenesis of PAH and may represent a possible therapeutic molecule to restrain the detrimental phenotype in the pulmonary vasculature.

### miR-34a in diabetes-associated vascular complications

Diabetes mellitus is a heterogeneous group of disorders that share hyperglycemia as main feature. The most important forms of Diabetes mellitus are type 1 (T1DM) and type 2 (T2DM) diabetes that have different metabolic characteristics and pathogenesis, nevertheless, they end up with similar long-term organs and tissues complications, including macro and micro vessels damage [7]. Indeed, VD, like atherosclerosis and medial calcification, are the principal reason of death and disability in diabetic patients [174]. Aging and diabetes lead to similar organ dysfunction that is driven by parallel molecular mechanisms, one of which is cellular senescence [175]. In young animal models of diabetes, hyperglycemia promotes the upregulation of a number of senescence-like features, particularly in vascular beds [176]. Accelerated vascular aging appears to involve a complex cascade of molecular events, named stress-induced premature senescence that culminate in the cellular acquisition of SASP [176]. This irreversible and deleterious process, which affects both ECs and VSMCs, results in upregulation of CDK inhibitors p21 and p16, altered expression of ECM proteins and of their degradative enzymes as well as the release of inflammatory molecules that contribute to the low-grade inflammatory status of diabetic patients [177]. Interestingly, similarly to aging, recent works also show that cellular elements with osteogenic phenotypes may take part to ectopic calcification in T2DM patients [178–180].

Epigenetic mechanisms, such as miRNAs, appear to play a key role in regulating cardiovascular dysfunction and anticipated vascular senescence in T2DM [181, 182]. Moreover, miRNAs are also considered valuable biomarkers of metabolic diseases. To this regard, in 2010 Kong et al. focused on the circulating expression patterns of 7 diabetes-related miRNAs, including miR-34a, during the onset of T2DM from susceptible to established T2DM in a cohort of Chinese patients [183]. In this study, miR-34a always showed the most significant differences between new identified T2DM patients compared with prediabetes and/or susceptible individuals with normal glucose tolerance [183]. In 2015 Zhu et al. performed a meta-analysis selecting miRNA expression profiling studies published between 1993 and 2014 involving T2DM patients in relation to non-diabetic individuals or animal models of diabetes with the aim to identify potential biomarkers of T2DM. They found 40 miRNAs significantly dysregulated in T2DM and among them miR-34a, miR-29a, miR-375, miR-103, miR-107, miR-132, miR-142-3p and miR-144 showed the potential as circulating biomarkers while, miR-199a-3p and miR-223 as potential tissue biomarkers of T2DM [184]. In 2017 Shen et al. found that miR-34a and miR-125b were upregulated in PBMC of patients with T2DM and positively or negatively correlated with plasma LDL/HDL and triglycerides/HDL ratio, respectively [185]. In 2019 Garcìa-Giacobo et al. evaluated the expression of numerous miRNAs, including miR-34a, in the sera of 82 pre-diabetic and T2DM Mexican patients. The data were correlated with β-cell functionality, insulin resistance, obesity, glycemia, dyslipidemia, diabetic foot and the presence of nephropathy or cardiovascular diseases [186]. The authors demonstrated that the level of circulating miR-34a, along with miR‐375 and miR‐146a, was not associated with β‐cell functionality. Nevertheless, their expression was differentially affected by glycemia, obesity, insulin treatment, and the presence of nephropathy and diabetic foot [186]. In 2016 Seyhan et al. assessed the plasmatic levels of 28 miRNAs in subjects with prediabetes, T2DM, T1DM and latent autoimmune diabetes of adults with the aim of identifying specific circulating pancreatic miRNAs as biomarkers of β-cell injury/dysfunction and diabetes subtyping. They found an upregulation of miR-34a in T2DM and T1DM subjects [187]. Similarly, in 2020 Banerjee et al. described increased levels of circulating miR-34a and oxidative stress markers in the plasma of T2DM middle-aged Indians ([188]; Table 2).

**Fig. 6:**
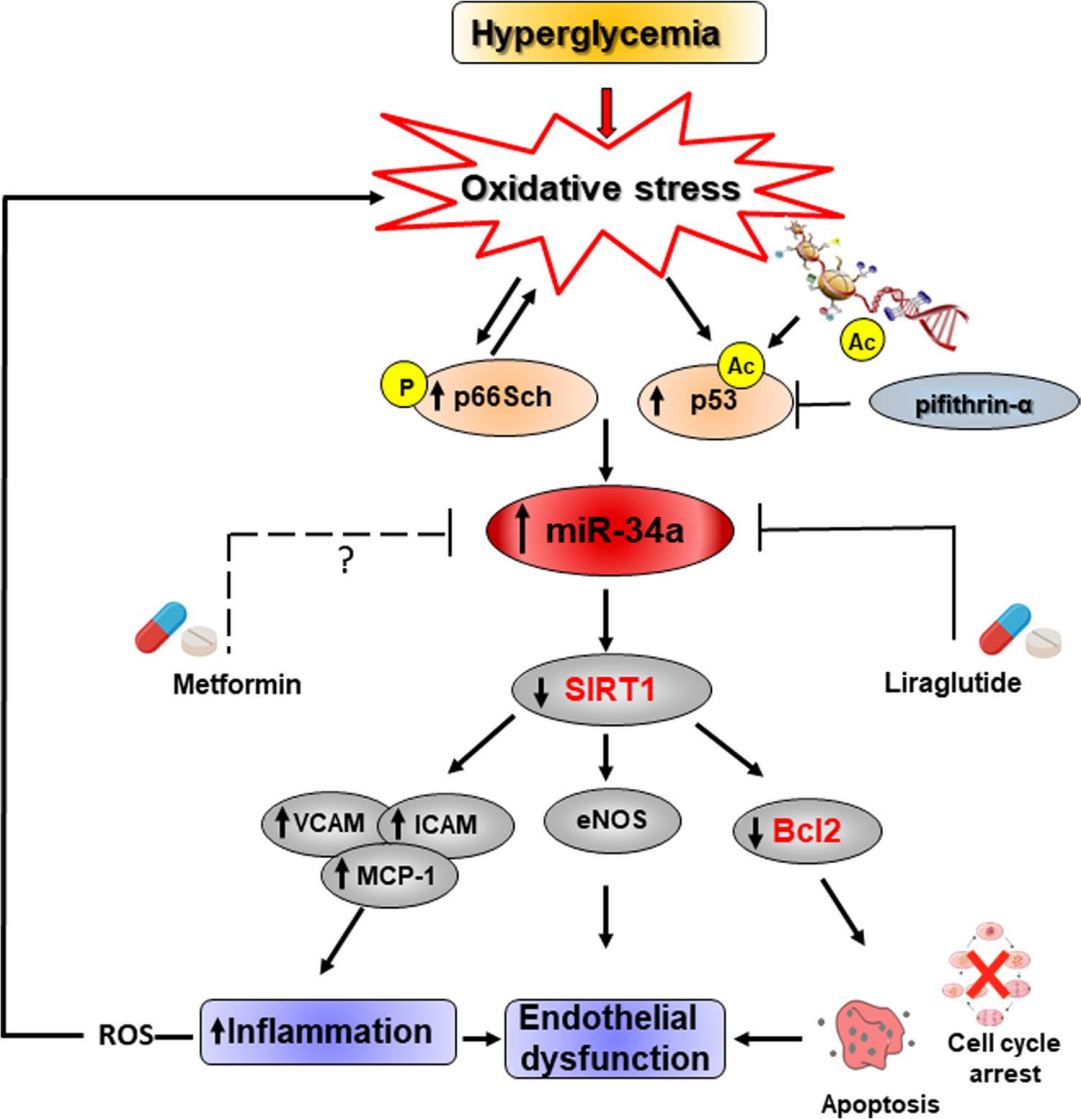
Molecular pathways triggered in vascular cells by miR-34a in diabetes. Hyperglycemia-induced oxidative stress promotes the acetylation of p53 transcription factor as well as p66Shc phosphorylation that are responsible for miR-34a upregulation and consequent reduced sirtuin 1 (SIRT1) and endothelial nitric oxide synthase (eNOS) expression in endothelial cells (ECs). SIRT1 downregulation, likely through NF-kB acetylation/stabilization, promotes Vascular Cell Adhesion Protein 1 (VCAM1), Intercellular Adhesion Molecule 1 (ICAM1) and monocyte chemoattractant protein-1 (MCP-1) protein expression leading to inflammation and endothelial dysfunction. This deleterious network contributes to intracellular reactive oxygen species (ROS) accumulation and cellular damage exacerbation. The p53-specific chemical inhibitor pifithrin‐α mitigates high glucose‐induced miR‐34a expression and SIRT1 downregulation. Glucose lowering agents, such as Metformin and Liraglutide, exert anti-aging, anti-inflammatory and vascular protective effects by miR-34a expression regulation

The importance of post-transcriptional regulation by miR-34a in the development of premature cardiovascular senescence in T2DM has recently emerged. The dynamic interplay among SIRT1, p66Shc, NF-kB, forkhead transcription factor (FOXO) and miR-34a has been described. SIRT1 is as key epigenetic regulator of p66Shc expression, a mitochondrial adaptor implicated in ROS accumulation, derangement of mitochondrial function, insulin resistance and diabetes [88, 189]. In physiological context p66Shc gene expression is blunted by SIRT1-induced histone de-acetylation that reduces the accessibility of transcription factors to chromatin. The loss of SIRT1 homeostasis promotes the activation of detrimental pathways involved in vascular aging [189]. Diabetes mellitus-dependent downregulation of SIRT1 results also in increased acetylation of nuclear factor NF-kB p65, and increased nuclear translocation and transcription of inflammatory genes [190]. Moreover, the inactivation of sirtuins in diabetes mellitus promotes FOXO 1, 3 and 4 acetylation with subsequent transcription of genes favoring cellular apoptosis, cell cycle arrest, accumulation of ROS and metabolic derangements [191, 192]. p53 is also highly expressed under diabetic condition and contributes to the pathogenesis of diabetes mellitus [193]. The acetylation of p53 stabilizes the protein and is essential for its function. Nuclear acetylated-p53 (ac-p53) activates the transcription of miR-34a in diabetes. Indeed, Wu et al. demonstrated that inhibition of p53 by direct silencing or the specific chemical inhibitor pifithrin‐α inhibited high glucose‐induced miR‐34a expression and miR‐34a-dependent SIRT1 reduction in murine aortic ECs. As a consequence, inflammation, in terms of VCAM, ICAM, MCP-1 gene expression, and ROS and lipid peroxides (malondialdehyde) production, was mitigated ([194]; Fig. 6). Pifithrin‐α as well as miR-34a-I injected diabetic mice exhibited a mitigated derangements of aortic ECs and VSMCs and tunica media thickness and impaired contraction and relaxation along with reduced SIRT1 and VCAM protein expression [194]. In 2016 Li and colleagues demonstrated that in type 2 diabetic db/db and streptozotocin-induced type 1 diabetes mice, systemic administration of LNA miR-34a inhibitor or specific endothelial miR-34a deletion is protective because it preserves endothelium-dependent vasorelaxation and SIRT1 levels. In this experimental context, the redox property of p66Shc was essential for miR-34a induction, whose expression was suppressed by antioxidants. They also showed that endothelial p53 was upregulated by high glucose, and p53 participated in miR-34a induction [195] (Table 1; Fig. 6). Arunachalam and colleagues investigated in mouse microvascular endothelial cells (MMECs) the molecular crosstalk between miR-34a, SIRT1 and the antidiabetic drug, metformin, in hyperglycemia-mediated impaired angiogenesis. They found that hyperglycemia-induced miR-34a had an anti-angiogenic action in MMECs via downregulation of SIRT1 and eNOS expression. Moreover, the expression of miR-34a was modulated by metformin demonstrating that miR-34a may represent a potential therapeutic target whereby this glucose lowering agent mediates its vasculoprotective actions and also for the prevention/treatment of diabetic vascular disease [33]. To this regard, numerous studies showed the metformin ability to modulate miR-34a expression, a pharmacological action that accounts for anti-aging and anti-inflammatory properties of the drug. However, the molecular mechanisms are still not completely understood and sometimes controversial [196, 197]. Interestingly, Zhang et al. showed that liraglutide, a glucagon-like peptide-1 analog belonging to the new-generation of glucose lowering agent, significantly improved aortic endothelial dysfunction in diabetic rats by downregulating key miRNAs, including miR-34a, and increasing the expression of anti-apoptotic protein Bcl2 and SIRT1 [198].

Taken together, these studies indicate that circulating levels of miR-34a associate with overt T2DM and T1DM, whereas its cellular expression is involved in the modulation of key diabetes mellitus-induced oxidant and inflammatory pathways (Fig. 6).

## Conclusion and future perspectives

Aging of the vascular wall and *inflammaging* are associated with a higher frequency of VD and their interaction with other risk factors (e.g., diabetes and hypertension), typical of old age, increases VD incidence. Thus, strategies aimed at delaying vascular aging and reducing *inflammaging* burn will prolong healthy lifespan. Recent findings highlighted that miR-34a, by directly affecting vascular aging/*inflammaging*, plays a causal role in the onset and progression of VD and therefore could represent a promising target for an effective strategy against them.

miR-34a increases with aging in the vasculature and induces VSMCs and ECs senescence and SASP, mechanisms that contribute to establish the systemic chronic low-grade inflammation (Fig. 1). We reported a positive correlation between circulating miR-34a and the SASP IL6, but not between miR-34a and IL8, in a population of healthy subjects ([35]; Table 1). Also, miR-34a induces IL6 but not IL8 expression and secretion in senescent VSMCs in vitro and in a mouse model of VC [35], suggesting that, at least in certain cell types, a causal relation among the two molecules exists that, eventually, can influence their systemic levels. A further characterization of miR-34a-related/modulated circulating SASP factors may lead to the identification of a specific signature associated with vascular aging/*inflammaging* and VD, helpful to establish individual biological age and new treatments for healthy aging.

miR-34a is also modulated by other VD risk factors, which promote vascular changes similar to aging (Fig. 1). Specifically, miR-34a upregulation contributes to aging- and VD risk factors-induced endothelial dysfunction by inhibiting ECs/EPCs proliferation and promoting their senescence through reduction of SIRT1 and NO bioavailability. miR-34a/SIRT1 axis also augments the expression of endothelial adhesion molecules that enhance leucocytes recruitment and arterial inflammation through NF-κB activation (Fig. 3). Notably, miR-34a is differentially modulated in VSMCs depending on the pathological milieu. miR-34a-induced senescence facilitates VSMCs mineralization and AAA and VC onset. Also, miR-34a promotes the secretion of inflammatory, matrix degrading and senescent inducers SASP factors in VSMCs that, in turn, spread senescence and calcification to nearby cells (Fig. 4). On the other hand, atherogenic stimuli and hypoxic conditions downregulate miR-34a levels in VSMCs and promote their proliferation and migration during plaque formation and pulmonary hypertension onset (Fig. 5).

Mechanistically, several points remain to be explored in order to gain knowledge about the molecular pathways modulated by miR-34a that affect ECs and VSMCs function in VD. Firstly, even though miR-34a can promote the SASP in both VSMCs and ECs, there are differences between the two cell types. In VSMCs, miR-34a-mediated SASP factors expression seems to be SIRT1 independent, whereas in ECs the downregulation of this sirtuin can partially mediate miR-34a-induced expression of VCAM1 and ICAM1 through enhanced acetylation of the RelA/p65 subunit of NF-κB [29, 34] (Figs. 3, 4). Hence, whether miR-34a promotes SASP factors expression in VSMCs by activating NF-κB or other pathways needs further investigations. Secondly, the signaling pathways activated by the pathological milieu responsible for miR-34a-mediated VSMCs phenotypic shifts are still poorly understood. DNA damage and the ATM serine/threonine kinase (ATM) signaling triggered by prelamin A accumulation have been shown as a key driver of VSMCs senescence, SASP and osteogenic differentiation [130]. In cancer cells, miR-34a regulates DNA damage response (DDR) and repair by targeting specific proteins including RAD51 [199]. It has been also shown that, in order to maintain vascular wall integrity during development, sustained Notch signaling is essential to preserve VSMCs contractile function and prevent reprogramming into chondrocytes [200]. Whether miR-34a regulates VSMCs senescence and osteogenic switch by influencing DDR and/or Notch1 pathway activation is of great interest. Finally, several findings report that the diabetic milieus induces miR-34a upregulation in ECs and impairs vascular function (Fig. 6); however, there are no data regarding the involvement of this miRNA in the process of VC associated with T2DM and consequent vascular complications [201].

miRNAs have emerged as a new class of drug targets providing new perspectives on the treatment of many diseases afflicting humans [202]. To date, several compounds, targeting or mimicking miRNAs, are currently tested in Phase I–II clinical trials [23]. Preclinical data in small animals have shown that the delivery of anti-miR-34a molecules are very effective to treat VDs (Table 2) underlying that therapies based on anti-miR-34a administration could be clinically relevant. Future studies on larger animals could represent the step forward to bridge preclinical evidences to clinical development. Certainly, possible side effects should be taken into account; for instance, considering the tumor suppressor activity of miR-34a, the chronic and systemic delivery of miR-34a inhibitors could promote tumorigenesis. In order to avoid this drawback, tissue local or cell-type-specific drug delivery systems could be settled.

Circulating miRNAs can be detected with high sensitivity and specificity and their levels have been found altered in patients with various diseases making them promising diagnostic and prognostic biomarkers [24]. As far as VD concerns, miR-34a levels have been found increased in the blood, atherosclerotic plaque and EPCs isolated from CAD patients and blood of subjects with hypertension and diabetes mellitus (Table 1). Nonetheless, further studies on tissues and plasma/serum samples on larger cohorts of patients are necessary to confirm the association between miR-34a and VD. Interestingly, preliminary works display the ability of some anti-lipidemic and -diabetic agents to modulate miR-34a expression. Specifically, atorvastatin decreases miR-34a levels in EPCs of CAD patients suggesting the involvement of miR-34a in the atheroprotective action of statins [145]. However, to date there are no data on the effect of this class of drugs on circulating miR34a levels in VD patients. Furthermore, liraglutide, a new glucose lowering agent, has shown to improve aortic endothelial dysfunction in diabetic rats by downregulating miR-34a [198]. Nevertheless, although promising, there are no clinical studies available to confirm the preclinical observations. Thus, the feasibility of miR-34a as a prognostic marker is promising and needs additional investigations.

In conclusion, additional studies are essential to broaden our knowledge on the mechanisms by which miR-34a affects vascular cells function during aging and in pathological conditions and to explore its therapeutic potential to counteract *inflammaging* and VD.

## Data Availability

Not applicable.
